# Performance of Basic Life Support by Lifeboat Crewmembers While Wearing a Survival Suit and Life Vest: A Randomized Controlled Trial

**DOI:** 10.3389/fpubh.2021.666553

**Published:** 2021-07-06

**Authors:** Allart M. Venema, Marko M. Sahinovic, Albert J. D. W. R. Ramaker, Yvette N. van de Riet, Anthony R. Absalom, J. K. Götz Wietasch

**Affiliations:** Department of Anesthesiology, University Medical Center Groningen, University of Groningen, Groningen, Netherlands

**Keywords:** resuscitation, basic life support, drowning, prehospital care, lifeboat

## Abstract

**Introduction:** Crewmembers of the “Royal Netherlands Sea Rescue Institution” (KNRM) lifeboats must wear heavy survival suits with integrated lifejackets. This and the challenging environment onboard (boat movements, limited space) might influence Basic Life Support (BLS) performance. The primary objective of this study was to assess the impact of the protective gear on single-rescuer BLS-quality.

**Material and Methods:** Sixty-five active KNRM crewmembers who had recently undergone a BLS-refresher course were randomized to wear either their protective gear (*n* = 32) or their civilian clothes (*n* = 33; control group) and performed five 2-min sessions of single rescuer BLS on a mannequin on dry land. BLS-quality was assessed according to Dutch and European Resuscitation guidelines. A between group analysis (Mann-Whitney U) and a repeated within group analysis of both groups (Friedman test) were performed.

**Results:** There were no major demographic differences between the groups. The protective gear did not significant impair BLS-quality. It was also not associated with a significant increase in the perceived exertion of BLS (Borg's Rating scale). Compression depth, compression frequency, the percentage of correct compression depth and of not leaning on the thorax, and ventilation volumes in both groups were suboptimal when evaluated according to the BLS-guidelines.

**Conclusions:** The protective gear worn by KNRM lifeboat-crewmembers does not have a significant influence on BLS-quality under controlled study conditions. The impact and significance on outcome in real life situations needs to be studied further. This study provides valuable input for optimizing the BLS-skills of lifeboat crewmembers.

## Introduction

Lifeboat-crews play an important role in the chain of survival for drowning victims as well as non-drowning victims in cardiac arrest (1–4). Early initiation of high quality basic life support (BLS) is a major link in the chain of survival (5–7). Therefore, all crew members are trained to perform BLS according to the latest guidelines of the Dutch and European Resuscitation Council (5, 6).

Before a lifeboat-crew can initiate BLS, they must first reach the victim. It can take longer to reach victims in the water than victims on dry land, since transport over water is slower than on land, especially when the weather conditions are bad (4). Once the victim is reached, several factors impact on the quality of BLS, such as fatigue and inadequate or decayed knowledge and skills (8–13). In addition, the challenging and sometimes dangerous environment on board a lifeboat such as (sudden) boat movements, unusual positions, fatigue and limited space, can also influence performance of BLS by crewmembers (1, 4, 14–16). Finally, lifeboat-crews are required to wear heavy protective equipment, which may also influence BLS-quality.

The “Royal Netherlands Sea Rescue Institution” (KNRM) is a maritime search and rescue organization that has provided help and assistance free of charge on open waters 24 h/day in all weather conditions since 1824 (2, 4). While on board a lifeboat, all crewmembers wear a waterproof survival-suit (protective gear).

The effects of wearing protective gear have been investigated for fire-fighters and show that work whilst wearing fire-fighter's protective gear consumes considerable extra energy (17). It has also been demonstrated that chest compressions can be less effective when the person performing the compressions is wearing personal protection equipment such as that used during chemical, biological, radiation or nuclear incidents (18).

These circumstances and protective gear however differ from the working environment lifeboat-crews encounter and the protective gear they wear. This topic is particularly relevant and topical to lifeboat crews dealing with drowning victims. It was recently highlighted in a review on behalf of the International Liaison Committee on Resuscitation (ILCOR), but at present there is a lack of scientific literature on this important topic for the drowning researchers community (19). With the results of the current study we hoped to gain new insights on the influence of wearing protective gear on BLS. This is also a necessary step in optimizing BLS performance by lifeboat-crews and enables rescue organizations to make evidence based decisions about BLS on lifeboats.

Identifying specific parameters for improvement can guide the optimization of the educational curriculum of lifeboat-crews of all maritime rescue organizations and as part of the KNRM quality management in particular. Such parameters might also lead to consideration of specialized modifications to the standard BLS-protocols for lifeboat-crews and might provide valuable input for templates like the Utstein Style For Drowning (USFD) (20).

The objective of this study was to investigate impact of the protective gear worn by KNRM lifeboat-crewmembers, on the quality of single-rescuer BLS-performance.

## Materials and Methods

The study was a randomized controlled trial performed in a simulation setting on land. The local ethics committee of the University Medical Center Groningen waived the requirement for a full consideration of the study on the basis that it does not fall within the remit of the Dutch law on medical research (Wet Medisch Onderzoek) (METc-reference number 2018/166). The study was registered with the Netherlands Trial Register (NL7324) according to ICMJE-requirements. Participants provided written informed consent prior to participation in this randomized controlled trial.

### Study Participants

The study participants were serving KNRM lifeboat-crewmembers. Via the KNRM headquarters the secretaries of 9 lifeboat-stations were sent an invitational e-mail explaining the study and requesting the participation of all crew-members. In total the crews of eight lifeboat-stations were willing to participate, after which a more detailed letter was sent to them to inform the individual subjects of the purpose and conduct of the study.

In order to be eligible to participate in this study, a subject had to be an active KNRM lifeboat-crewmember (including trainees/aspirants) of 18 years or older, provide written informed consent and have passed a BLS refresher course prior to the measurements of this study to eliminate differences of skill deterioration after training as much as possible (12). Due to logistical reasons, it was not feasible to provide the refresher course the day prior to the measurements for all participants. It was thus decided to allow a maximum period of 1 to 21 days between the refresher course and the measurements.

KNRM members who were not part of a lifeboat-crew (office members, inspectors, etc.), were (possibly) pregnant, or had physical disabilities were excluded from participation.

### Study Procedure

**Figure 1:**
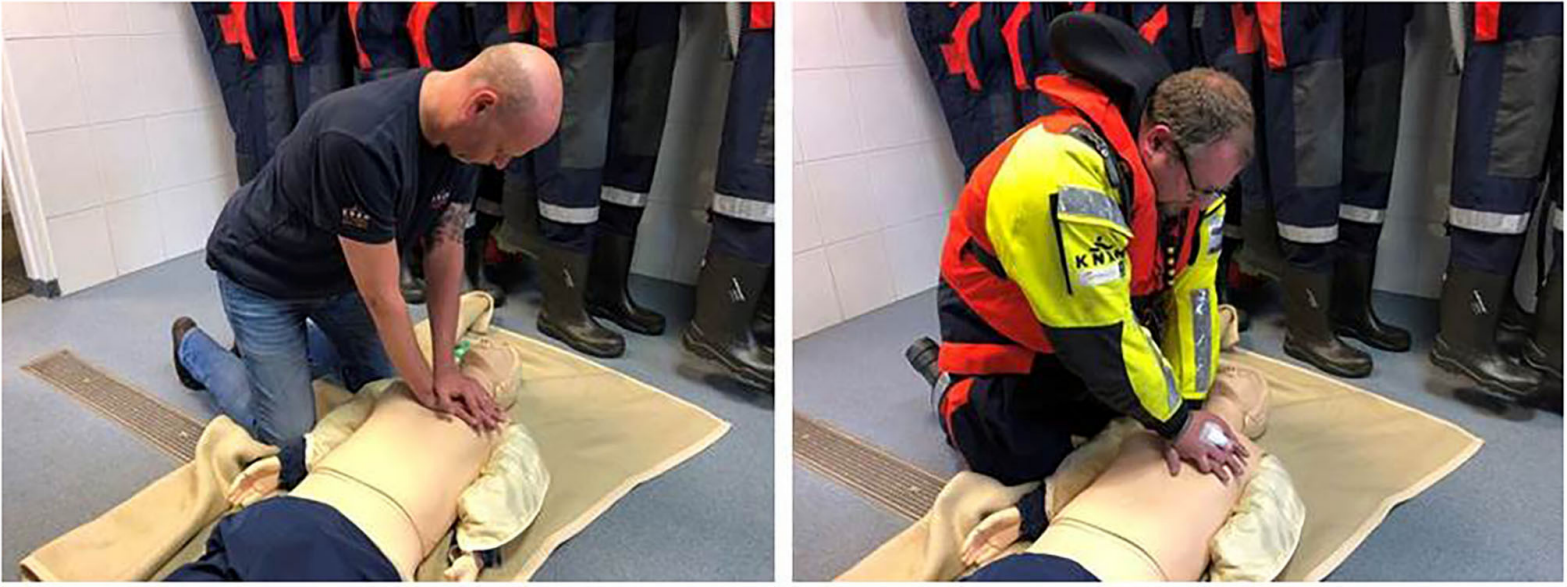
Two lifeboat crewmembers performing BLS on a mannequin.

Before the start of the measurements, the subjects received a further verbal explanation of the study and were asked to sign an informed consent form. Subsequently block randomization was performed (sealed envelope technique) to create two groups. The participants in the study group wore protective gear: a waterproof survival-suit with a sealed collar, arm-cuffs and boots, of only modestly flexibility with an insulating inner part and an integrated life jacket. When dry, they weigh between 11 and 13 Kg, depending on the size, and between 13 and 16 Kg when wet. (Survitec SuvivalOne, Type 2111 GP000 A or GP001 A, Survitec Group Limited, London, United Kingdom). Participants in the control group wore only their normal civilian clothes (Figure 1). All volunteers were subsequently asked to fill in a short questionnaire about demographical data, physical fitness using the metabolic equivalents score (MET-score) by means of The Veterans Specific Activity Questionnaire (translated in Dutch) and their expectations about the potential influence of the protective gear on BLS performance (21).

The standard number of crewmembers on a lifeboat depends on the type of lifeboat and ranges from 3 to 6. The coxswain steers the lifeboat but needs a navigator to bring the vessel to the correct location. This means that in the worst-case scenario when only three crewmembers are on board a lifeboat during a resuscitation, only two crewmembers are available to perform BLS, and they do so in turns usually lasting 2 min each. This scenario was simulated during the study. The measurements were conducted on land to eliminate potential confounds caused by differences in wave and weather conditions causing different boat movements.

The individual participants in both groups performed single-rescuer BLS on a resuscitation-mannequin with electronic data collection (Resusci Anne CPR-Mannequin with skill reporter software; Laerdal Medical, Stavanger, Sweden) during five sessions of 2 min. All participants participated in the study at their own lifeboat station (indoors). As per their usual training, mouth-to-mask ventilations were provided, but via a standard airway filter device attached to the mask. After every 2-min session of BLS the participants had a 2-min break during which they were asked to complete the Borg's rating scale ranging from 6 (no exertion) to 20 (maximal exertion), which is a validated tool to describe perceived exertion (22). During the performance of BLS, (observed by an advanced life support instructor), no feedback was provided.

After their fifth BLS-session, all volunteers were given the opportunity to provide additional written comments on how realistic they found the performance of BLS during this study to be.

### Variables

Quality of BLS was judged by measuring compliance and performance relative to published standards as follows: mean compression depth 50-60mm, mean compression frequency 100–120/min, percentage compressions for which compression depth was correct: 100%, percentage of not leaning on the thorax after compressions (i.e., full thoracic recoil) 100%, percentage time during which hand placement on the thorax was correct: 100%, ventilation frequency <10/min, mean ventilation volume of recue breathing 500–600 ml; hands off time (time of no-BLS) as short as possible (6, 23, 24). These parameters were automatically recorded using Laerdal skill reporter software (Laerdal Medical, Stavanger, Sweden).

We also assessed the differences between the groups regarding demographic factors including Body Mass Index (BMI), and physical fitness (Metabolic Equivalent of Task (MET) score), function and experience on lifeboat, and prior BLS-experience on the quality of BLS. In addition, we assessed if the perceived exertion of the rescuers after each BLS session were congruent with the quality of BLS. Finally we sought to assess the subjective opinions of rescuers on the influence of protective gear on their performance of BLS, and on how realistic they thought the simulation was.

### Statistical Analysis

The study data was entered in a database and subsequently checked by two persons (AMV and MMS), after which statistical analysis was performed using SPSS Statistics version 23 (IBM, New York, United States). We used the Kolmogorov-Smirnov test to assess the distribution of the data. Normally distributed data are summarized as mean (SD) per group, and were tested for statistical significance between groups using the students *t* test. Non-normally distributed data are summarized as median (IQR) per group, and tested for statistical significance between groups using the Mann-Whitney U test. The Friedman test (a non-parametric repeated measurement test) was used to analyze differences in the repeated measurements within both groups. We considered a *p* ≤ 0.05 to be statistically significant.

### Sample Size Calculation

As far as we know, there are no published data on the influence of the wearing of protective gear that lifeboat-crewmembers wear with respect to the quality of BLS. This led us to choose a pragmatic approach in which the number of crewmembers per lifeboat-station (i.e., the maximum potential number of participants), potential drop-outs and feasibility considerations (discussed with KNRM representative) were taken into account. It was not possible to include all KNRM lifeboat-crewmembers, because of the additional time costs related to the study. On this basis it was concluded that the maximum number of participants that could be included without jeopardizing the successful conclusion of the study, was 60 (i.e., 30 per group).

## Results

**Figure 2:**
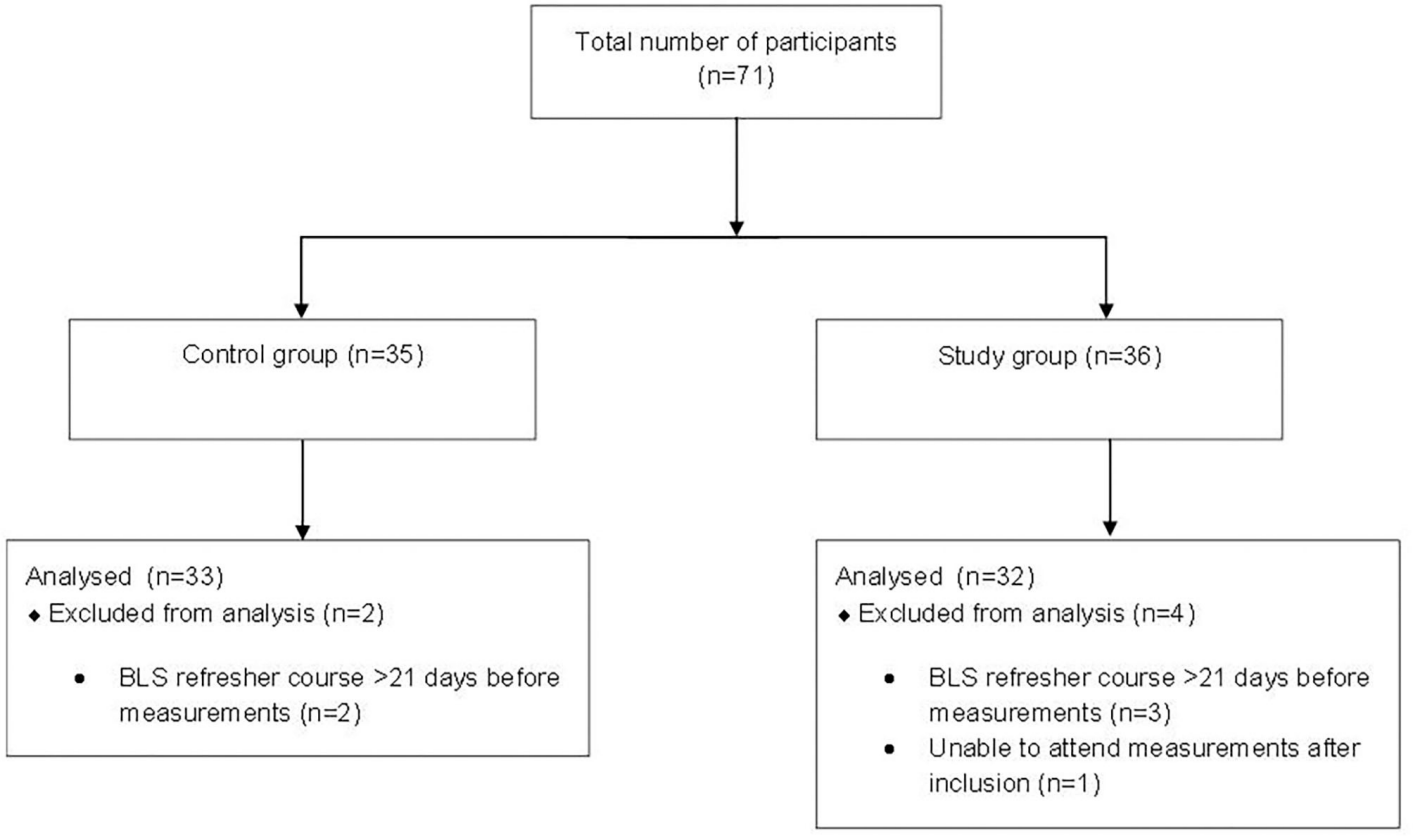
Inclusion flow diagram.

Between October 23, 2018 and July 18, 2019 in total 71 volunteers from eight lifeboat-stations participated in the study. The data of 6 crewmembers were excluded from analysis (Figure 2). As seen in Table 1 the demographics of the study group (*n* = 32) and the control group (*n* = 33) were similar. The majority of participants in both groups were male and the median ages of participants in the control and study group were 39 and 41 years, respectively.

**Table 1:** Demographics.

	**Control group (*N* = 33)**	**Study group (*N* = 32)**
**Gender** ***n*** **(%)**		
Male	31 (93.9)	31 (96.9)
Female	2 (6.1)	1 (3.1)
**Mean age in years (SD)**	39 (9.6)	41 (8.8)
**Mean body weight in Kg (SD)**	92.2 (11.1)	88.2 (13.0)
**Mean height in cm (SD)**	185.6 (6.5)	182.1 (7.3)
**Mean Body Mass Index (SD)**	26.8 (3.1)	26.6 (3.3)
**Occupation** ***n*** **(%)**		
Medical	1 (3.0)	0
Non-medical	32 (97.0)	31 (96.9)
Not reported	0	1 (3.1)
**Median MET score (IQR)**	10.0 (9.0–12.5)	10.0 (9.0–12.0)
**Years active as crewmember** ***n*** **(%)**		
0–4 years	11 (33.3)	16 (50.0)
5–10 years	10 (30.3)	6 (18.8)
>10 years	11 (33.3)	10 (31.3)
Not reported	1 (3.0)	0
**Median number of days between BLS refresher course and measurements (IQR)**	14.0 (7.0–14.0)	14.0 (7.0–14.0)
**Real BLS experience reported** ***n*** **(%)**		
Yes, on Land	7 (21.2)	8 (25.0)
Yes, on lifeboat	2 (6.1)	1 (3.1)
Yes, on Land and on lifeboat	4 (12.1)	3 (9.4)
No	18 (54.6)	17 (53.1)
Not reported	2 (6.1)	3 (9.4)
**Expected influence protective gear on BLS quality** ***n*** **(%)**		
Yes, positive influence	0	0
Yes, negative influence	16 (48.5)	18 (56.3)
No, no influence	8 (24.2)	7 (21.9)
Don't know/ no opinion	8 (24.2)	7 (21.9)
Not reported	1 (3.0)	0
**Opinion volunteers on how realistic measurement session was** ***n*** **(%)**		
Very realistic	1 (3.0)	2 (6.3)
Realistic	11 (33.3)	14 (43.8)
Neutral	13 (39.4)	11 (34.4)
Unrealistic	7 (21.2)	4 (12.5)
Very unrealistic	0	0
Multiple answers	1 (3.0)	1 (3.1)

The medians and IQRs of the primary study parameters per epoch, as well as the medians of all epochs combined, are shown in Tables 2–4. Between-group comparisons per epoch as well as the medians of the combined epochs show that with exception from the “percentage of correct thoracic hand placement” in epoch 2 (*p* = 0.049) there were no significant differences.

**Table 2:** BLS performance related to thoracic compressions of both groups.

	**Control group (*n* = 33)**	**Study group (*n* = 32)**	***P* (Mann-Whitney *U*)**
**Mean compression depth in mm [median (IQR)]**			
Epoch 1	49.0 (44.5–58.0)	51.5 (48.0–57.8)	
Epoch 2	47.0 (42.5–56.5)	50.5 (44.5–56.8)	
Epoch 3	48.0 (43.0–56.0)	51.0 (44.3–56.0)	
Epoch 4^*^	49.0 (41.0–55.0)	51.0 (45.0–56.0)	
Epoch 5	46.0 (40.0–55.0)	49.5 (42.5–54.8)	
Median value all epochs	48.0 (42.5–55.5)	51.0 (44.8–56.8)	0.379
Friedman χ2(*df* = 4) (p)	14.6 (0.006)	19.9 (0.001)	
**Mean compression frequency [median (IQR)]**			
Epoch 1	108.0 (97.5–122.5)	116.5 (100.3–122.8)	
Epoch 2	113.0 (105.5–122.5)	115.0 (101.3–127.8)	
Epoch 3	114.0(106.0–124.5)	112.5 (105.5–129.8)	
Epoch 4^*^	117.0 (107.5–126.5)	113.0 (108.0–129.0)	
Epoch 5	119.0 (106.5–125.5)	116.0 (106.5–129.0)	
Median value all epochs	114.0 (105.5–125.0)	113.5 (105.3–129.0)	0.793
Friedman χ2(*df* = 4) (*p*)	47.5 (<0.001)	28.5 (<0.001)	
**Percentage of correct compression depth [median (IQR)]**			
Epoch 1	38.0 (4.0–99.0)	72.0 (29.8–99.0)	
Epoch 2	28.0 (1.0–98.5)	72.0 (11.0–99.0)	
Epoch 3	18.0 (0–99.0)	69.0 (4.8–98.5)	
Epoch 4^*^	42.0 (0–99.0)	80.0 (7.0–98.0)	
Epoch 5	8.0 (0–97.5)	69.0 (2.8–98.0)	
Median value all epochs	38.0 (0.0–98.5)	69.5 (8.4–98.8)	0.372
Friedman χ2(*df* = 4) (p)	5.9 (0.208)	11.3 (0.024)	
**Percentage of not leaning on the thorax [median (IQR)]**			
Epoch 1	84.0 (31.5–96.5)	65.0 (13.5–95.5)	
Epoch 2	72.0 (26.0–97.5)	51.0 (6.5–98.0)	
Epoch 3	84.0 (36.0–98.0)	43.0 (10.5–99.0)	
Epoch 4^*^	71.0 (32.0–93.5)	32.0 (6.0–98.0)	
Epoch 5	71.0 (29.0–96.5)	31.0 (7.0–99.0)	
Median value all epochs	72.0 (25.0–96.5)	47.5 (10.5–99.0)	0.244
Friedman χ2(*df* = 4) (p)	2.0 (0.745)	2.1 (0.725)	
**Percentage correct thoracic hand placement [median (IQR)]**			
Epoch 1	100.0 (100.0–100.0)	100.0 (92.5–100.0)	
Epoch 2^†^	100.0 (100.0–100.0)	100.0 (70.5–100.0)	
Epoch 3	100.0 (100.0–100.0)	100.0 (47.5–100.0)	
Epoch 4^*^	100.0 (100.0–100.0)	100,0 (82.0–100.0)	
Epoch 5	100.0 (100.0–100.0)	100.0 (83.8–100.0)	
Median value all epochs	100.0 (99.5–100.0)	100.0 (70.5–100.0)	0.246
Friedman χ2(*df* = 4) (*p*)	5.0 (0.284)	8.9 (0.065)	
**Hands off time in seconds (time of no-CPR) [median (IQR)]**			
Epoch 1	7.0 (6.0–8.5)	7.5 (6.0–9.0)	
Epoch 2	7.0 (6.0–8.5)	8.0 (6.0–8.8)	
Epoch 3	7.0 (6.0–8.0)	7.0 (6.0–8.8)	
Epoch 4^*^	7.0 (6.0–8.0)	7.0 (6.0–8.0)	
Epoch 5	7.0 (6.0–8.0)	7.0 (6.0–8.0)	
Median value all epochs	7.0 (6.0–8.0)	7.0 (6.0–8.0)	0.692
Friedman χ2(*df* = 4) (*p*)	7.0 (0.137)	3.9 (0.418)	

* *Results of one volunteer in study group not recorded due to equipment failure in epoch 4*.

† *p = 0.049*.

**Table 3:** BLS performance related to mouth to mask ventilations of both groups.

	**Control group (*n* = 33)**	**Study group (*n* = 32)**	***P* (Mann-Whitney *U*)**
**Ventilation frequency [median (IQR)]**			
Epoch 1	8.0 (8.0–10.0)	9.5 (8.0–10.0)	
Epoch 2	9.0 (8.0–10.0)	10.0 (8.0–10.0)	
Epoch 3	10.0 (8.0–10.0)	10.0 (8.0–10.0)	
Epoch 4^*^	10.0 (8.0–10.0)	10.0 (8.0–10.0)	
Epoch 5	10.0 (8.0–10.0)	10.0 (8.0–10.0)	
Median value all epochs	10.0 (8.0–10.0)	9.5 (8.0–10.0)	0.426
Friedman χ2(*df* = 4) (*p*)	21.0 (<0.001)	3.5 (0.479)	
**Mean ventilation volume of recue breathing in ml [median (IQR)]**			
Epoch 1	858.0 (601.0–1031.5)	715.5 (522.8–1054.5)	
Epoch 2	911.0 (604.0–1129.0)	782.5 (602.0-1252.3)	
Epoch 3	1,015.0 (587.0–1189.5)	783.0 (611.5-1246.8)	
Epoch 4	1,011.0 (620.0–1207.5)	951.0 (669.0–1341.0)	
Epoch 5	1,077.0 (586.0–1209.5)	981.0 (650.5–1191.3)	
Median value all epochs	1,023.0 (586.5–1186.0)	807.0 (611.0–1233.8)	0.733
Friedman χ2(*df* = 4) (*p*)	15.6 (0.004)	39.3 (<0.001)	

* *Results of one volunteer in study group not recorded due to equipment failure in epoch 4*.

**Table 4:** The Borg's rating scale after each BLS epoch of both groups.

	**Control group (*n* = 33)**	**Study group (*n* = 32)**	***P* (Mann-Whitney *U*)**
**Borg's Rating scale [median (IQR)]**			
Epoch 1	11.0 (10.0–11.0)	11.0 (11.0–11.0)	
Epoch 2	11.0 (11.0–12.0)	11.0 (11.0–12.0)	
Epoch 3	12.0 (11.0–13.0)	11.0 (11.0–13.0)	
Epoch 4	12.0 (11.0–13.0)	12.0 (11.0–13.0)	
Epoch 5^*^	12.0 (11.0–13.0)	12.0 (11.0–13.0)	
Median value all epochs	11.0 (11.0–13.0)	11.0 (11.0–13.0)	0.868
Friedman χ2(*df* = 4) (*p*)	40.6 (<0.001)	44.1 (<0.001)	

* *Borg score of 1 person in study group and 2 persons in control group not recorded in epoch 5*.

The Friedman test demonstrated a significant within-group difference over time for both groups for the parameters “mean compression depth” (decrease), “mean compression frequency” (increase), “volume of rescue breathing” (increase) and the “Borg's rating scale” (increase). Given the small sample size, and the small changes, we did not perform *post hoc* pairwise analyses of the findings of different epochs. A significant within-group difference over time of the parameters “ventilation frequency” (increase) and “percentage of correct compression depth” (decrease) was only detected for one group (control group and study group, respectively) (Tables 2–4).

In total six volunteers (all in the study group) provided comments regarding the warmth of the protective gear. In both groups the majority of the volunteers expected that the protective gear would have a negative influence on BLS quality. Both groups considered the study measurements realistic, or had a neutral opinion about it.

## Discussion

Overall, the results of this study do not demonstrate a significant negative influence of the protective gear worn by KNRM lifeboat-crewmembers, on the quality of BLS-performance. The results also do not show a significant increase in the perceived exertion as measured on the Borg's Rating scale. However, for both groups compression depth, compression frequency, the percentage of correct compression depth, not leaning on the thorax and ventilation volumes were suboptimal when evaluated according to the BLS guidelines (6).

To our knowledge, this was the first study specifically designed to investigate the influence of the protective clothing worn by lifeboat-crewmembers on the quality of BLS. Research aimed at improving the quality of resuscitation at sea is of great importance to the KNRM and other maritime rescue organizations which seek to optimize the quality of their efforts.

Due to the current COVID-19 pandemic, increasing attention has been focussed on the personal protective equipment (PPE) worn by rescuers. As a consequence, aquatic rescue organizations around the world are re-evaluating their resuscitation protocols, seeking on the one hand to avoid contamination of rescuers as much as possible, while on the other hand assuring high quality resuscitation (25–27). The results of the current study may be of interest to rescue organizations, as they do not demonstrate a profound negative effect of the heavy survival suits with life jackets on BLS quality.

Interestingly, the results stand in contrast with the expectation of most volunteers in this study who believed that the protective gear has a negative influence. While there were tendencies toward some parameters being different in volunteers wearing protective gear, these differences did not reach statistical significance (the “mean compression depth” tended to be greater, the “percentage of correct compression depth” tended to be higher, and ventilation volumes tended to be smaller but still too high). There can be several reasons for this discrepancy. Firstly, the sample size was pragmatically chosen and was small, which may have limited the power of the study to detect significant differences.

Secondly, the KNRM volunteers are all used to working in their protective gear, potentially masking negative effects by for example increased heat production when wearing protective-gear. During our study, participants performed BLS for a total of 10 min each, and so it is possible that if BLS is performed for a longer time, then the protective clothing will have a greater effect on performance. On the other hand, in practice it seems that in most cases more than two persons are available to perform BLS. Finally, the measurements were made indoors on land. It is possible that the additional challenges of working on a moving lifeboat, especially during bad weather, might further amplify any possible effect of the clothing on BLS performance. However, especially during bad weather, the conditions at sea itself will probably influence BLS performance as well. This is supported by a recent study by Duncan et al. that demonstrated the negative effects of simulated wave motions on search and rescue tasks and maintaining balance (28). Although BLS performance was not tested in their study, rough seas might also affect BLS performance.

Physical fitness has also been demonstrated to have an effect on BLS performance. As the MET and Borg rating scores were similar in our groups of participants and 2-min BLS intervals are deemed feasible, we do not expect that physical fitness would have influenced the results of our study (15, 29). Nonetheless, it is possible that physical fitness might be of importance onboard a lifeboat in real life, especially in bad weather conditions.

These mechanisms, as well as the effects of the influence of protective-gear on patient outcome, need further investigation. It may therefore be useful to include one or more variables about protective gear worn by lifeboat-crewmembers and other aquatic rescuers resuscitation efforts in future updates of the recommended USFD-dataset. This might improve the granularity and applicability of data recorded in future studies (20).

There was a broad variability in the performances of the individual volunteers, despite the fact that they all had passed a recent BLS refresher course. Prior studies have shown that decay of skills after BLS courses occurs within 3 to 6 months (12). Therefore, before performing the measurements, all volunteers in our study were required to have passed a BLS refresher course in advance to minimize the effects of skill deterioration. Since both groups in this study had the same median value of seven days for this timeframe and because this was well within the three-month period, we do not expect it to have influenced the results. The BLS refresher course currently is an annual item on the KNRM curriculum. Perhaps more frequent, short, BLS refresher moments could help in reducing this large variability and increasing overall BLS-performance (30).

A recent report of a study by Seesink et al. describes the performance of BLS and the use of automated external defibrillators (AED) in real-life situations by KNRM lifeboat-crews by using the data recorded electronically by AED's (4). Their results show a median compression frequency of 120/min (IQR: 111–131) and a median total BLS time of 32 min (IQR 25–45 min). The compression frequencies we recorded in our study are thus comparable with real-life circumstances. Similar results were found in a different simulation setting, involving helicopter rescue swimmers wearing a dry suit and life jacket (31). In this latter study BLS performance during a helicopter flight was comparable to that of BLS on land, and similar issues concerning the quality of BLS as in our study were present.

Volunteers in our study group leaned more on the thorax and compressed the thorax slightly deeper than the volunteers of the control group, which may have been caused by the extra weight of the protective gear. Although these differences did not reach statistical significance, the higher incidence of leaning on the thorax may well be clinically relevant as it impairs the extent of thoracic recoil which may influence the effectiveness of BLS performance. It is also possible that the control group had poorer BLS skills than the study group, since the volunteers in this group administered more excessively high ventilation volumes in combination with more superficial chest compressions. This is however unlikely as participants were randomized to the control and study group, the control group participants scored better on not leaning on the thorax, and there were no differences between the groups in their demographics and in the time since the last BLS training. It is therefore more likely that these differences were caused by the absence of the protective gear in this group.

As no previous studies have investigated the influence of the protective gear worn by lifeboat-crewmembers, the sample size of this study was pragmatically chosen. As explained in the Methods section, a larger sample size for this study was also not feasible. Should it be possible for other groups to access a larger sample size, then our data could inform the calculation of sample size.

Although the use of mannequins helps to create a safe study environment, avoids interference with rescue and resuscitation operations and eliminates potential negative effects on patients in cardiac arrest, it's not the same as a real resuscitation. However, since the results in part correspond with real life circumstances and most crewmembers considered the setting in which the measurements were done to be realistic or had a neutral opinion about it (Table 1), we expect the results to be as close to reality as possible for a mannequin study (4).

We did not collect temperature-related data during this study, even though some of the volunteers commented on the warmth when wearing the protective gear. As both groups performed BLS under the same conditions, we do not expect this to have influenced the results of this study.

## Conclusions

This study shows that the survival suits with integrated life jackets worn by KNRM lifeboat-crewmembers do not have a significant influence on the quality of BLS performance under controlled study conditions. The impact and significance on outcome in real life situations needs to be studied further. The results provide valuable input for optimizing the BLS skills of KNRM lifeboat-crewmembers and other organizations involved in nautical search and rescue.

## Data Availability Statement

The datasets presented in this article are not readily available. We are happy to share the data with the editor and reviewers. However, as the participants only consented to their data being used for the purposes of this study, we cannot make it publicly available.

## Ethics Statement

Ethical review and approval was not required for the study on human participants in accordance with the local legislation and institutional requirements. The local ethics committee of the University Medical Center Groningen waived the requirement for a full consideration of the study on the basis that it does not fall within the remit of the Dutch law on medical research (Wet Medisch Onderzoek) (METc-reference number 2018/166). The study was registered with the Netherlands Trial Register (NL7324) according to ICMJE-requirements. Participants provided written informed consent prior to participation in this randomized controlled trial.

## Author Contributions

AV, AA, and JW were involved in the conception and design of the study. AV, AR, YR, and MS were involved in the data collection. AV, AA, JW, and MS were involved in the analysis and interpretation of the data. All authors have been involved in drafting the manuscript or revising it critically for important intellectual content. All authors have given final approval of the version to be published.

## Conflict of Interest

AV reports non-financial support from Laerdal Medical (the resuscitation mannequins and corresponding software used for the purpose of this study were provided free of charge by Laerdal Medical, who were not involved in the conduct and reporting of this study), during the conduct of the study; and AV is a volunteer at one of the KNRM lifeboat stations. AA reports personal fees and other from The Medicines Company, personal fees and other from Philips, personal fees from Janssen Pharma (Johnson and Johnson), grants and personal fees from Carefusion (BD), personal fees from Ever Pharma, personal fees from Orion, personal fees from PAION, other from Rigel Inc., outside the submitted work. The remaining authors declare that the research was conducted in the absence of any commercial or financial relationships that could be construed as a potential conflict of interest. The handling editor declared a shared committee/research group with one of the authors AV at time of review.
